# Use of Self-Measured Blood Pressure Monitoring to Improve Hypertension Equity

**DOI:** 10.1007/s11906-022-01218-0

**Published:** 2022-08-24

**Authors:** Elaine C. Khoong, Yvonne Commodore-Mensah, Courtney R. Lyles, Valy Fontil

**Affiliations:** 1 Division of General Internal Medicine at Zuckerberg, Department of Medicine, San Francisco General Hospital, UCSF, Building 10, Ward 13, 1001 Potrero Avenue, San Francisco, CA 94110 USA; 2 UCSF Center for Vulnerable Populations at Zuckerberg San Francisco General Hospital, San Francisco, USA; 3 Johns Hopkins School of Nursing, Baltimore, MD USA; 4 Department of Epidemiology, Johns Hopkins Bloomberg School of Public Health, Baltimore, MD USA

**Keywords:** Hypertension, Blood pressure, Disparities, Self-measured blood pressure, Telemonitoring

## Abstract

**Purpose of Review:**

To evaluate how self-measured blood pressure (SMBP) monitoring interventions impact hypertension equity.

**Recent Findings:**

While a growing number of studies have recruited participants from safety-net settings, racial/ethnic minority groups, rural areas, or lower socio-economic backgrounds, few have reported on clinical outcomes with many choosing to evaluate only patient-reported outcomes (e.g., satisfaction, engagement). The studies with clinical outcomes demonstrate that SMBP monitoring (a) can be successfully adopted by historically excluded patient populations and safety-net settings and (b) improves outcomes when paired with clinical support. There are few studies that explicitly evaluate how SMBP monitoring impacts hypertension disparities and among rural, low-income, and some racial/ethnic minority populations.

**Summary:**

Researchers need to design SMBP monitoring studies that include disparity reduction outcomes and recruit from broader populations that experience worse hypertension outcomes. In addition to assessing effectiveness, studies must also evaluate how to mitigate multi-level barriers to real-world implementation of SMBP monitoring programs.

## Introduction

Disparities in hypertension continue to be a major challenge in public health contributing significantly to mortality worldwide [1, 2]. Within high-income countries, persons with low-income, including those with Medicaid insurance within the USA, are more likely to have uncontrolled blood pressure (BP) and its associated morbidity and mortality [3–6]. In the USA, racial and ethnic minority groups have lower rates of BP control with Black patients frequently having the highest prevalence and experiencing worse outcomes compared to all other racial/ethnic populations [1, 6–8]. There are also geographic and neighborhood-level disparities in the burden of hypertension. Individuals who live in rural areas in the USA are more likely to develop hypertension [9]. Hypertension is also growing in importance in low- and middle-income countries (LMICs), where the prevalence of hypertension has risen significantly [2]. This growing prevalence is particularly concerning since only 8% of people residing in LMICs have their BP controlled, which contributes to the higher rates of morbidity from hypertensive heart disease [2, 10]. While these data refer to essential hypertension, similar patterns also appear for pregnancy-related hypertensive disorders [11–13].

Factors contributing to hypertension disparities occur at the level of the community/neighborhood, healthcare system, and individual. Community factors such as access to healthy food or underinvestment in rural areas or neighborhoods with predominantly non-white populations are associated with higher blood pressure in individuals who live in those neighborhoods [14–16]. These community factors (e.g., unsafe built environment, poor public transportation options, closing of low profit clinical settings) cause individual behaviors, such as limited physical activity, high salt diet, or poor adherence to scheduled appointments or prescribed medications that also impact hypertension management [17, 18]. Limited health literacy or educational attainment also inhibit patient activation, which is crucial to self-management of a chronic disease like hypertension [19].

Although these socio-economic factors contribute to poor outcomes, hypertension disparities are by no means inevitable or static. They can be made better or worse by the way we deliver care. Healthcare process factors that are controlled or influenced by the health system, such as infrequent follow-up and suboptimal prescribing of medication treatment for hypertension, are major barriers to achieving BP control [20–22]. For example, health systems that have implemented team-based care where non-physician team members can make treatment decisions, standardized treatment algorithms, and frequent follow-up have achieved improved BP control across their patient population regardless of race or income [23–29]. Conversely, we have noted the potential for an increase in hypertension disparities during the COVID-19 pandemic when access to in-person clinic visits was limited and telehealth was not equitably accessible or usable by historically underserved populations or the safety-net systems that care for them [30–32].

## The Importance of Out-of-Office Blood Pressure Measurements

Several scientific societies and professional organizations have recommended out-of-office BP (OOBP) measurement to confirm the diagnosis of and manage hypertension [33–35]. OOBP measurements include all BP measurements that are taken outside the clinical setting. For instance, the 2017 Hypertension Clinical Practice guidelines recommends the use of OOBP measurements to confirm the diagnosis of hypertension and treat hypertension (Class 1 recommendation, Level of Evidence A) [34]. OOBP measurements help to identify BP phenotypes including masked hypertension and white-coat hypertension [36, 37]. Masked hypertension is defined as not having high office BP measurements and having high out-of-office BP values. Conversely, white coat hypertension is defined as having high office BP measurements and not having high OOBP values.

OOBP include ambulatory blood pressure monitoring (ABPM), which is an automated technique of recording BP, often over a 24-hour period. Although ABPM is strongly associated with cardiovascular disease (CVD) related events, this OOBP technique is poorly-tolerated by patients due to interference with daily activities and sleep [38]. Moreover, ABPM requires provider training and is not widely available in primary care practices. In contrast, many OOBP measurements are collected similar to in-office blood pressure measurements by either the patient, a caregiver, community health worker, or healthcare professional. Although there have been studies that have focused on collection of OOBP measurements by community health workers or healthcare professionals at community settings (e.g., churches or barbershops) [39, 40], we limit this review to focus on self-measured blood pressure (SMBP) monitoring, or home blood pressure monitoring, when patients (or their caregivers) measure the patient’s blood pressure out of the office. As with all OOBP measurements, SMBP readings are more prognostic of CVD, stroke, and target organ damage than office BP readings [41–43]. Although we include telemonitoring interventions (in which telecommunication tools are used to transmit data for clinician review) in this review, we will primarily use the term SMBP as many studies do not involve remote transmission of data, and telemonitoring has not been proven to be more effective than SMBP with clinical support [44].

Published meta-analyses have demonstrated that SMBP monitoring in combination with co-interventions including team-based care, regular support from trained clinicians, antihypertensive medication titration, lifestyle counseling, and/or patient education is associated with significant improvement in BP and reduction of BP [42, 43, 45–49]. Distribution of blood pressure monitors without any of these additional supports is less effective at improving blood pressure control [26]. Yet, the integration of SMBP in clinical care to provide the necessary co-interventions is inconsistent and fragmented [50••]. Furthermore, the health disparities impact of SMBP monitoring remains unclear [51].

During the COVID-19 pandemic, there has been a rapid expansion in SMBP monitoring and the integration of SMBP monitoring into telemedicine. Telemedicine use increased rapidly across health systems to address gaps in clinical care and mitigate the spread of COVID-19. Although telemedicine has been highlighted as a potential strategy to address disparities in health care access, there is also an acknowledgment that digital literacy challenges and disparities in device and broadband access may further exacerbate health disparities [31]. However, telemedicine may be further enhanced and increased in safety by the integration of remote patient monitoring including SMBP and clinical support to improve lifestyle, medication adherence, and BP control [52••, 53]. SMBP monitoring in the context of telemedicine also has the potential to address disparities in hypertension control by addressing transportation barriers that are associated with missed visits and poor BP control [54, 55]. The transmission of SMBP readings and other clinical information from the patient’s home to the clinical team is associated with improvement in clinical outcomes including BP reduction, and optimization of antihypertensive therapy as well as patient-reported outcomes such as self-management and activation [54, 56].

## How SMBP Monitoring Could Address Equity in Hypertension Control

As noted above, many factors contribute to disparities in hypertension control. SMBP monitoring programs are particularly well-positioned to address inequities in hypertension outcomes that result from disparities in access to in-person clinical care or patient engagement.

Historically excluded populations disproportionately face time or transportation barriers to accessing brick and mortar clinical settings that are only open during work hours. For example, low-income low-wage workers may be employed at multiple jobs without the ability to take time off for a healthcare visit; patients who rely on public transit or reside in rural areas may spend hours commuting to and from an office visit. These barriers result in fewer clinical visits and therefore fewer blood pressure measurements and opportunities to identify uncontrolled hypertension [57, 58]; missed visits can result in disparities in hypertension outcomes [20]. SMBP monitoring facilitates equity through earlier identification of uncontrolled hypertension and provides more opportunities for clinicians to intensify medications in response to uncontrolled blood pressure values.

SMBP monitoring programs also provide opportunities to address disparities in patient activation. By engaging in SMBP, patients are empowered to understand and recognize how individual behaviors (medication adherence, diet, or exercise behaviors) impact blood pressure control. Though these individual behaviors play only a small role in equity, SMBP facilitates increased patient engagement and may facilitate improved medication adherence, disease understanding, and lifestyle behaviors as well as nudge clinicians into providing more timely treatment [59].

Below, we discuss several issues related to SMBP monitoring and equity. We first start off with a narrative review of the literature to describe what we know and do not know about how SMBP monitoring interventions can improve hypertension equity. Due to differences in healthcare system infrastructure in high-income countries versus LMICs, we focus on studies conducted within high-income countries. Based on these studies, we then describe the issues related to implementing SMBP monitoring interventions in a way that ensure equity. We conclude with recommendations to advance the utility of SMBP monitoring interventions to mitigate hypertension disparities.

## What is Known About the Impact of SMBP Monitoring on Equity

Despite the potential for SMBP programs to address hypertension equity, there is limited literature on whether it improves equity. A 2021 guide from the United States Centers for Disease Control and Prevention [51] notes that while SMBP with clinical support is highly effective, there is insufficient knowledge about its impact on health disparities. This is not surprising since a 2012 paper from the Agency for Healthcare Research and Quality focused on research needs in SMBP made no mention of evaluating the impact of SMBP on equity [60].

To better understand how SMBP impacts equity, we reviewed studies that met one of the following criteria: (a) ensured adequate inclusion (or target recruitment of) patient populations that experience worse hypertension outcome or (b) explicitly reported outcomes focused on disparity reduction in key clinical or process outcomes. Within Table 1, we highlight a few SMBP studies that have centered equity. Although we used these broad criteria to focus on literature related to equity in SMBP, there have been an insufficient number of large, high-quality studies to reach any definitive conclusions. Instead, research in this area is nascent and often includes pilot studies with limited sample size and where the primary outcome is related to feasibility. However, from these pilot studies and the handful of larger, high-quality studies, there are some broad themes.

**Table 1:** Self-measured blood pressure monitoring studies focused on equity

How study focused on equity	Study aims	Study outcomes/key findings	Author
Patient population 525 hypertensive adults in six primary care practices in Lenoir County, eastern rural North Carolina; ~ 60% Black; ~80% household income < 40,000 Primary outcome: SBP change stratified by race (African-American vs white)	Evaluate impact of multicomponent practice-based quality improvement (QI) intervention (practice-level and patient-level interventions) on lowering SBP on cohort of patients with uncontrolled hypertension	- Significant decrease in SBP at 12 months with no difference between white or African-American participants	Cené et al. [76•], 2017
Patient population/setting 159 Black patients at an urban primary care clinic in Baltimore, MD	Compare effectiveness of community health worker (CHW) intervention with home BP monitor vs CHW with extra training in shared decision making or problem-solving	- Significant improvements in BP control and SBP in all three arms	Boulware et al. [64], 2020
Patient population/setting: 206 patients with pregnancy-related hypertension from a Philadelphia, PA hospital; ~ 65% Black; majority Medicaid insured Outcome: explicit measurement of disparity	Compare text-based vs in-person care for monitoring blood pressure postpartum	- Higher rates of blood pressure measured in first 10 days postpartum in text-message group - Text-messaging resulted in 50% reduction in racial disparity in blood pressure ascertainment in comparison to in-office usual care	Hirshberg et al. [61•, 106], 2018
Patient population/setting: 900 patients from eight NY community health centers/clinics; ~ 25% Black; ~ 65% Hispanic; > 50% uninsured/self-pay	Evaluate effectiveness of provision of home blood pressure monitor on BP control	- SMBP without clinical support did not improve control over usual care	Yi et al. [70•], 2015
Patient population / setting: 217 low-income, rural patients (~ 55% Black) from six rural primary care practices in North Carolina Outcome: explicit measurement of disparity	Evaluate for differential patterns of treatment intensification between Black and white patients enrolled in SMBP program	- Similar rates of medication intensification between races - Reduction in mean SBP after intensification greater in white patients than Black patients	Cummings et al. [65•], 2019
Patient population/setting: 120 low-income, rural patients (~ 60% Black) from a Mississippi academic medical center	Evaluate the feasibility, safety, and effectiveness of home BP telemonitoring with remote hypertension management	- Compared to propensity-matched controls, intervention participants had greater reductions in SBP and DBP at six-months	Clark et al. [75], 2021
Patient population/setting: 823 Medicaid insured patients in Texas	To characterize adherence of Medicaid patients with hypertension to daily telemonitoring	- Approximately 40% were described as nonadherent and transmitted data < 60% of time even after reminder calls - SBP improved among all participants regardless of adherence, but adherent participants had greater SBP improvements	Park et al. [74•], 2021

Most SMBP-related studies that have focused on hypertension equity have done so by either prioritizing recruitment of disadvantaged populations or conducting the study in health centers or settings that disproportionately care for patient populations that experience worse health outcomes. Many pilot studies have specifically focused on racial/ethnic minority patient populations, with the greatest number of studies focused on improving hypertension control among Black patients [61•, 62–64, 65•, 66–69, 70•, 71]. There are a smaller number of studies that included Latinx populations [66, 70•, 72], and there is a dearth of studies in Asian, Pacific Islander, or Indigenous populations [73]. Despite the high prevalence of uncontrolled hypertension in lower socioeconomic status or rural populations, there have only been a handful of published studies focused on these populations [65•, 74•, 75, 76•].

We found that many equity-focused SMBP monitoring studies reported engagement in monitoring programs or adherence to monitoring activities. Consistent with other digital health interventions for hypertension management [56], equity-focused SMBP studies also found patient interest and ability to engage in remote blood pressure monitoring programs [77•]. Few studies have explicitly evaluated for a reduction in disparities in clinical outcomes, or even the process outcomes that are the mechanisms through which SMBP monitoring could improve equity, such as medication adherence, medication intensification, or blood pressure ascertainment. Without better information on process outcomes, we do not have sufficient understanding of the mechanisms through which improved clinical outcomes are achieved; without information about why specific interventions work, who they work for, and in what settings they work, we will be limited in our ability to achieve equity. As noted by advocates for equity in cardiovascular disease, there is a need to bring an implementation science lens into this field [78].

Along these lines, many studies that have explicitly centered equity have paired self-monitoring interventions with other intervention components (such as community health workers or medication reminders). While these types of multi-component interventions are often more successful in improving disease outcomes, it makes it difficult to ascertain how much improved clinical or process outcomes can be attributed to SMBP monitoring versus other components of the intervention.

Although some studies have conducted practice-level analyses or support for bringing SMBP into routine clinical care [76•, 77•], studies have disproportionately focused on individual-level analyses and addressing patient-facing barriers to SMBP adoption [79•]. As noted in the National Institute on Minority Health and Health Disparities Research Framework, disparities manifest in several levels of influence (e.g., individual, interpersonal, community) [80, 81]. Most studies we found focused on SMBP as a patient-level intervention without addressing other levels of influence (notably clinical teams and healthcare systems) that are necessary to support effective SMBP implementation, especially when adoption is needed in safety-net systems that may have challenges to adopting new innovations [82].

We identified few studies that explicitly measured the impact of monitoring programs on disparities. Hirshberg et al. [61•] described how a SMBP program reduced disparities in blood pressure ascertainment in postpartum patients. Cummings et al. [65•] evaluated for disparities in medication intensification after implementation of hypertension telemonitoring data. Cené et al. evaluated the impact of a multi-component quality improvement intervention on systolic blood pressure (SBP) change in white versus Black participants [76•]. (See Table 1 for more details about these studies.)

While most studies reported the impact of these programs on clinical or process outcomes, there was rarely specific evaluation of disparity reduction. Although improvement in clinical/process outcomes is important, if the difference in these outcomes is not measured between the advantaged and disadvantaged population, it is possible that the improvement in the population experiencing disparities is smaller than the advantaged population, thereby widening the disparity. This phenomenon has been called an intervention-generated inequity and is a common occurrence for digital health interventions, particularly since digital health tools may be less accessible to populations that experience disparities in health outcomes [83]. Many of the studies focused on equity also exclusively recruited historically excluded populations [64, 65•, 68, 69, 72, 74•]. While greater inclusion of marginalized and historically excluded populations in clinical trials is necessary [84], it is difficult to know the impact of any implemented intervention on disparity reduction if the more advantaged population is not also included in the study.

Lastly, studies focused on SMBP used a variety of communication modalities to return the blood pressure values back to the clinical team, including telephone phone calls, short-messaging system (SMS)/text messaging, mobile phone applications, and patient portals [85]. Frequently, the choice of communication modality was designed with feedback from participants to ensure accessibility for the population. There were no clear findings on the effectiveness of one communication tool over another; specifically, no studies indicated greater effectiveness of using telemonitoring approaches (where data transmission occurs automatically and digitally) over SMBP with telephonic or text-messaging–based communication of blood pressure values.

## Forthcoming Studies About SMBP Monitoring and Equity

Given the growing interest in SMBP monitoring, we also wanted to understand the research questions being explored in yet to be published studies. In Table 2, we describe trials registered on ClinicalTrials.gov that were identified based on searching for hypertension monitoring trials focused on equity or disparities. We had hoped these upcoming or recently completed trials would begin to advance knowledge in the areas outlined above. However, we found that upcoming trials continue to largely replicate prior gaps in the literature.

**Table 2:** Upcoming, ongoing, and recently completed SMBP monitoring trials

Clinical trial name	Patient population	Intervention component	Primary outcomes
Actions to Decrease Disparities in Risk and Engage in Shared Support for Blood Pressure Control (ADDRESS-BP) in Blacks [87]	Black adults receiving primary care at one of 20 primary care practices affiliated with New York University	Practice facilitation to support implementation of three multi-level evidence-based interventions: nurse case management, home BP monitoring, and use of a community health worker	Rates of BP control; Implementation costs; Incremental cost effectiveness ratio; Practice facilitation
A Cardiometabolic Health Program Linked with Clinical-Community Support and Mobile Health Telemonitoring to Reduce Health Disparities (LINKED-HEARTS) [107]	Adults identifying as non-Hispanic White, non-Hispanic Black/African American and/or Hispanic	A multi-level project that intervenes at the practice level by linking home blood pressure monitoring (HBPM) with a telemonitoring platform (Sphygmo). The program incorporates team-based care by including community health workers (CHWs) and pharmacists to improve the outcomes of multiple chronic conditions	Blood pressure control
Home Blood Pressure Telemonitoring LINKED With Community Health Workers to Improve Blood Pressure (LINKED-BP) [108]	Adults identifying as non-Hispanic White, non-Hispanic African-American, or Hispanic	A multi-level intervention that includes a telemonitoring application (Sphygmo), SMBP and community health workers to prevent hypertension	Change in systolic blood pressure
Blood Pressure Improving Control Among Alaska Native People (BP-ICAN) [109]	Adults identifying as Alaska Native or American Indian who have received care at participating Alaska clinics	Participants receive a home BP monitor to be used twice daily for 12 months; participants will also receive text messages with educational, motivational, and reminder messages	Change in SBP at 12 months; Frequency of medication adjustment
OPtimizing Technology to Improve Medication Adherence and BP Control (OPTIMA-BP) [110]	African-American patients > 50 years old with hypertension	A multi-component technology enabled intervention including web-based education sessions, medication management app, home BP monitoring, and nurse counseling	BP control at 6 months; Health Related Quality of Life at 6 months
Use of an Innovative Mobile Health Intervention to Improve Hypertension Among African-Americans [111]	Sixteen African-American patients with hypertension receiving primary care at participating Federally Qualified Health Centers	FAITH! HTN app that promotes self-management through education modules; home BP monitor that syncs to the app; CHW	BP change; Participant engagement with self-monitoring; Hypertension self-care
Video-based Intervention to Address Disparities in Blood Pressure Control After Stroke (VIRTUAL) [112]	Adult patients with recent stroke and diagnosis of hypertension	Early follow-up after a stroke via telehealth with a multidisciplinary team, remote blood pressure monitoring, and medication adjustment by a pharmacist	6-month blood pressure control

Specifically, while there continues to be a focus on Black patients, other patient populations (e.g., rural, Indigenous, Asian, Pacific Islander, or Medicaid insured) that experience hypertension disparities have poor representation (or unknown representation if this information is not collected). Moreover, no studies have proposed measuring a reduction in disparity as a primary or secondary outcome for clinical or process outcomes. All these interventions included SMBP monitoring with several other components, and it is unclear if studies will evaluate which components of each intervention are most responsible for any changes in outcomes. For example, are improved clinical outcomes driven primarily by digital communication, or is human-coaching and intensive in-person support a crucial aspect to improved outcomes?

Fortunately, there are upcoming studies focused on how to implement SMBP in real-world clinical care. The importance of this evaluation has been brought to light by the COVID-19 pandemic and the well-documented challenges safety-net systems faced in implementing remote patient monitoring tools [86]. For example, the ADDRESS-BP trial plans to provide practice facilitation to support implementation of SMBP monitoring (and other evidence-based interventions) and is evaluating both effectiveness and implementation outcomes [87]. This attention to real-world implementation is encouraging and should be continued in other trials.

## What we Still Need to Learn About SMBP Monitoring and Equity

Given the early literature in this space and still limited number of future studies in this space, there are many areas that require further investigation to understand the impact of SMBP monitoring programs on hypertension equity. We outline three broad areas for future study.

### Better Defining and Broadening Evaluation of Populations that Experience Hypertension

There is a need for larger, high-quality studies that include a greater diversity of participants that experience worse hypertension outcomes. While there are a growing number of studies focused on Black/African–American participants, there are fewer focused on other racial or ethnic groups that experience worse hypertension outcomes, including Latinx, Pacific Island, Indigenous, and some Asian ethnicities. Furthermore, more studies should evaluate interventions in populations with low income (or Medicaid insured as a proxy) and limited educational attainment. Unfortunately, this information is not always collected by researchers during clinical trials. Despite the nearly 10% disparity in hypertension control between rural and urban populations, studies are more frequently conducted in urban areas where most research centers are based.

### Explicitly Evaluating Disparities in Clinical and Process Outcomes

As noted above, while it is crucial that equity studies focus on BP outcomes or key process outcomes (e.g., medication adherence, medication intensification) that result in BP change, it is also necessary to explicitly measure the disparities between populations. Without measurement of the disparity between populations, it is difficult to know if interventions will advance equity. We acknowledge that it can be challenging (although very possible) to power a primary outcome based on disparity reduction; however, at the very least, researchers with an eye towards equity should at least plan to evaluate and measure the impact of interventions on disparities. This therefore requires researchers to recruit from both the advantaged and disadvantaged populations; moreover, to power a clinical trial on a primary outcome of disparity reduction, research trials will need to include a large enough sample size from both populations.

### Using Implementation Science to Understand Mechanisms that Improve Equity and how to Support Implementation

It is also necessary to advance understanding about how best to implement remote monitoring interventions to advance equity. Using implementation science tools in SMBP monitoring studies would improve understanding of (1) which intervention components work for which patients, and (2) how best to implement remote monitoring programs in under-resourced clinical settings that disproportionately care for patients that experience hypertension disparities. Due to the multicomponent nature of many SMBP monitoring interventions (especially when focused on populations that experience disparities), researchers need to closely evaluate which component of these interventions drive change in outcomes. Moreover, evaluation of clinic or community-level levers to support implementation is needed. Although some trials are beginning to conduct pragmatic evaluations of SMBP monitoring as part of clinical care, there is wide variation in clinical settings and SMBP monitoring implementation [77•, 85]. More studies focused on implementation in diverse health systems and patients will inform understanding of how SMBP monitoring programs can be designed to ensure equity. In particular, instead of conducting studies only in the clinical setting, for historically excluded populations that face challenges in accessing healthcare system, it can be beneficial to partner with community-based organizations to support patients in SMBP monitoring [88••].

## Multi-Level Implementation Considerations to Facilitate Equity

As implementation scientists, we want to highlight this last point about evaluating implementation outcomes to ensure equity. While it is necessary to evaluate the efficacy of SMBP monitoring on reducing disparities, it is also crucial to explore ways to facilitate effective and equitable implementation of SMBP monitoring interventions. Findings in real world settings are frequently different from clinical trial results and conducting trials in diverse settings can be helpful for providing insights on the effectiveness (rather than efficacy) of interventions. For example, the study team involved with the Hirshberg et al. study on SMBP for postpartum patients found that when they implemented their intervention at a second site, the percentage of their study population that identified as Black decreased from 70% to 40%. Fortunately, the authors still found that in real-world implementation, there was similar improvement in process and clinical outcomes between their Black and non-Black participants [62]. However, this study underscores that even if SMBP monitoring interventions improve equity in a study setting, real-world SMBP implementation may unintentionally worsen disparities.

**Fig. 1:**
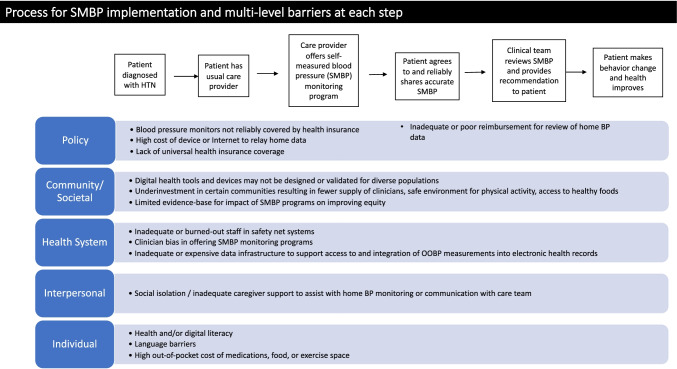
Multi-level barriers to process for SMBP implementation

We are particularly concerned about unintended consequences because as with other digital health tools, there are multiple levels of barriers to implementation that need to be considered to ensure equity [89, 90]. Figure 1 depicts a simplified process for a patient to participate in an SMBP monitoring program; even with this simple depiction, it is clear that there are numerous steps involved in the workflow of implementing an SMBP monitoring program. Many steps are needed to engage patients in self-monitoring at home, such as the matching of devices to patient needs and skills as well as the motivation and opportunity to sustain home monitoring in the midst of everyday life. Similarly, on the clinical team side, there are new workflows needed that often require multiple care team members to (1) recommend/encourage monitoring at home; (2) review the home BP readings from patients (whether in real-time or at a regularly scheduled interval); and (3) provide/enact clear and timely treatment plans based on the home BP readings.

Within this multi-step process, there are possibilities for drop-off or gaps along the entire spectrum, from onboarding to routine clinical management incorporating home BP data. These barriers to adoption of this digital health intervention can occur at multiple levels [89–91]. For example, individuals from marginalized communities or facing communication barriers within the healthcare system experience barriers at policy levels (such as cost of Internet at home to participate in SMBP programs or access only to devices that do not meet patients’ needs), interpersonal levels (such as the need for social support or assistance in using devices or communicating with their care team), and individual levels (such as differences in language accessibility or health literacy) [92].

It is well known that patients from racial/ethnic minority backgrounds and those with low-income are more likely to receive healthcare from public healthcare or safety net healthcare settings in the USA, and therefore barriers to healthcare systems must also be considered [93]. Safety-net settings are less likely to have robust digital infrastructure in place to easily facilitate remote monitoring programs, such as electronic health records that can integrate with remote BP monitors to ingest data [94–96]. In addition, these settings are often understaffed, which can limit their ability to shift team members to manage home BP readings and/or communicate with patients remotely about care plans [97].

Moreover, both patients/families and clinical care teams need digital tools that will meet their needs and preferences, rather than having to continuously adapt tools not designed for their use for their setting [98]. At a community/societal level, technology and device developers need to consider all patients and clinical systems when developing digital tools. For example, before the COVID-19 pandemic, only OOBP values collected through telemonitoring were eligible for pay for performance programs. This therefore required the patient to have the skills and access to devices and Internet to sync their BP monitor with a mobile device or computer that would relay the blood pressure values to the healthcare system, which would also have needed to invest in software that integrated remote data with their electronic health record. The barriers to this workflow were innumerable and understandably adoption of SMBP monitoring was very low.

In addition, as with other digital health tools, many organizations have failed to develop SMBP programs that support patients who speak languages other than English and are available in digital formats that are easy to use (e.g., do not require Bluetooth connections or complex mobile apps) [99]. Investing in tools for diverse populations is critical to address disparities in uptake and use. At a basic level, it is crucial that blood pressure devices are validated in diverse populations to ensure accuracy in diverse populations [100, 101]. This is not just an academic consideration; a study among Alaska Native and American Indian research participants specifically identified trust in device performance in their community as an important consideration in encouraging SMBP monitoring adoption [100].

Lastly, as is clear from the discussion above, the available evidence about SMBP is not often centered on these patients or settings. For example, in addition to randomized controlled trials, it is critical to collect real-world evidence on engagement and adherence to SMBP across patient groups and in multiple settings to advance our understanding of how best to implement these programs. Similarly, all studies should routinely document key characteristics of their sample related to equity, such as use and effectiveness of SMBP by race/ethnicity, gender, age, language, socioeconomic status, and health and digital literacy [102].

## The Path Forward for Stakeholders to Ensure SMBP Monitoring Improves Equity

Given the promise of SMBP monitoring programs to improve hypertension control, but the real challenges to equitable implementation, we provide the following recommendations to key stakeholders to ensure that SMBP implementation can improve equity.

### Research Funders and Researchers

Researchers and research funders have a clear role to play to advance equity in SMBP implementation. As noted in the section on what we still need to learn, funders and researchers need to (a) both better define the populations that experience hypertension disparities and broaden evaluations to include more of those populations; (b) explicitly conduct subgroup analyses to evaluate the impact of intervention implementation on disparities; (c) conduct implementation-focused studies that increase understanding of how to implement these programs in real-world settings and which components of multi-component interventions are most important for specific populations. Although specific study designs and methodologies are beyond the scope of this review, we encourage funders and researchers to utilize pragmatic hybrid effectiveness-implementation clinical trials [103] to address and prioritize these implementation considerations.

Another key aspect to facilitating development of a useful evidence-based is ensuring that researchers collect all relevant sociodemographic traits (such as income, educational attainment, and digital literacy) to better understand for which patients these programs work. While some funders require reporting of the age, gender, and race/ethnicity of anticipated research participants, there is little enforcement of these planned targets during the recruitment process. Also, funders should provide a larger budget and longer timeline that acknowledges the additional time, effort, and resources to recruit historically excluded populations (e.g., translation of consent documents, relationship-building with trusted community-based organizations). Moreover, there should be consideration of expanding what sociodemographic traits (e.g., language, income, literacy, insurance status/coverage) are collected from participants to understand the applicability of research findings to marginalized populations.

While there are areas for future research, there are key steps that other stakeholders can take now to increase equitable implementation of SMBP.

### Policy Makers and Payors

Policy makers and payors need to acknowledge the innumerable barriers that patients and healthcare systems face to implement a successful SMBP programs. At a basic level, increasing access to healthcare and health insurance will improve equitable access to SMBP monitoring programs across the USA. Specific to SMBP monitoring, payors should not return to pre-pandemic policies that required SMBP values to be transmitted digitally (i.e., telemonitoring) for clinicians to receive “credit” for pay-for-performance metrics or reimbursement. Given both the patient-facing challenges of using telecommunication tools for remote patient monitioring and health system challenges (especially in safety net systems) of integrating these data into electronic health records, it would be inequitable to force use of only telemonitoring to improve hypertension outcomes, especially since studies have not demonstrated the superiority of telemonitoring.

Despite no proven superiority of telemonitoring, we recognize that many healthcare systems are moving towards digital communication of BP values. Many patients face structural barriers to accessing the devices or high-quality Internet access to utilize these telemonitoring tools. Policy makers should pursue policies that increase access to low-cost digital devices and internet access and increase investment in infrastructure that makes high-quality internet accessible to all communities. Similarly, if SMBP programs rely on apps or other digital health tools, regulatory agencies can build in baseline accessibility requirements into their approval processes to address equity. For example, as the U.S. Federal Drug Administration (and similar agencies in other countries) begin approving digital therapeutic tools, there could be requirements related to digital platform usability and language access.

For all SMBP programs, payors can also address cost-related barriers for patients by reimbursing for BP monitoring devices. Harmonization of policies from all payors (within the USA, this includes private insurance, Medicaid, Medicare, and Medicare Advantage) would facilitate equity. At this time, not all payors have the same policies which is confusing for both patients and clinical teams; continued reimbursement for telemedicine visits must be paired with reimbursement for patient monitoring tools (such as BP monitors) that support high quality telemedicine visits. Moreover, with an eye on equity, payors should strongly consider reimbursing for a wider variety of BP monitors, including BP monitors with extra lurge cuffs or BP monitors that ease communication of values back to clinical teams (e.g., cellular-enabled BP monitors that allow for data transmission without advanced digital literacy skills). Reimbursing for monitors that meet all patients’ needs may help ensure SMBP monitoring produces equitable improvements in clinical outcomes.

To address the disproportionate burden that safety-net systems face in supporting marginalized patients to use digital tools, payors need to consider how to provide reimbursement or compensation for the additional work conducted by the clinical team to support patient monitoring at home. For example, US payors should continue moving away from fee-for-service payment models and towards value-based payment, alternative payment models, and other approaches where clinical teams can choose the best method to care for a patient that may include more or less in-person care based on the patients’ needs. Payors can also center equity by prioritizing pay-for-performance metrics that are focused on disparity reduction.

### Digital Tool Developers

Digital tool developers need to begin to specifically innovate for historically excluded populations. This involves including these patient populations in design conversations to ensure that tools are usable by patients with limited health literacy, limited digital literacy, or with communication barriers (such as a non-dominant language preference). Tools should also be validated in diverse populations, as studies have shown that ignoring diverse populations clearly leads to disparities and inaccuracy in clinical decisions [104, 105].

While conversations about digital design equity have often focused on patient-facing barriers, we want to highlight that digital tools should also consider challenges faced by clinicians in underresourced safety-net settings. Given the importance of both patients and clinicians in successful implementation of SMBP monitoring, any digital tool used to facilitate SMBP monitoring should be designed to work well for safety-net systems that have fewer financial resources to pay for expensive software or hardware, are more likely to use electronic health records with fewer advanced features, and are more likely to be understaffed. Therefore, it is crucial to design tools that can be easily integrated into clinical team workflows and structure in safety net systems.

### Health System Leaders and Clinical Teams

Front-line clinicians and healthcare delivery systems also can take direct steps to ensure equitable access to SMBP monitoring programs. Clinicians should recognize that some patients may need additional support to adopt and use these tools and be prepared to provide training to patients as needed. If there is an opportunity to choose different BP monitors or data submission platforms, clinicians should choose products with greater usability. For example, by selecting a cellular-enabled blood pressure monitor that allows automatic uploading of BP values for clinician review rather than asking patients to sync via a Bluetooth device or manually entering values into a patient portal. Clinicians should also consider avoiding programs that require patients to have advanced digital literacy skills, which poses unnecessary additional barriers to equitable patient participation.

Given the higher proportion of hypertension among patients with low-income, clinical teams should learn about payor coverage for BP monitors so they can help patients secure home BP monitors. While payors can make this easier for clinical teams by having similar coverage, in the meantime clinical teams should be prepared to support patients with acquiring BP monitors by developing explicit workflows for acquiring BP monitors and teaching patients how to use these devices.

Healthcare system leaders must also recognize that collecting and responding to home blood pressure values requires significant clinical team time. Instead of expecting clinical teams to do this work between office visits, healthcare systems that adopt these programs should create a team-based infrastructure that will optimize the chances of successful implementation. Investing in hardware, software, personnel, or culture change that makes this work sustainable for clinical teams will help ensure accessibility and equal treatment for higher-needs patients. These investments may include: selecting electronic health records and other software with advanced features to facilitate data integration and usable digitally connected care; reallocating personnel to robustly support population health panel management; and optimizing performance reporting and billing processes to capture reimbursement available with current policies to secure funding that sustains the infrastructure required to provide equitable SMBP programs.

## Conclusions

SMBP monitoring is a well-known approach to improve hypertension control, but its impact on equity is understudied and largely unknown. Early studies are promising and suggest that SMBP can be successfully adopted by historically excluded patient populations and implemented in community health centers and safety-net settings. Future research needs to include better representation from more populations that experience worse hypertension outcomes; explicitly evaluate the impact of SMBP monitoring programs on disparities between patient populations; and evaluate both effectiveness and implementation outcomes to inform real-world implementation of SMBP monitoring. There is a role for all stakeholders — including funders, researchers, policy makers, payors, digital tool developers, and healthcare teams — to advance implementation of SMBP monitoring to improve hypertension equity.

## References

[1] Rethy L, Shah NS, Paparello JJ, Lloyd-Jones DM, Khan SS (2020). Trends in hypertension-related cardiovascular mortality in the United States, 2000 to 2018. Hypertension.

[2] Dai H, Bragazzi NL, Younis A, Zhong W, Liu X, Wu J, Grossman E (2021). Worldwide trends in prevalence, mortality, and disability-adjusted life years for hypertensive heart disease from 1990 to 2017. Hypertension.

[3] Shahu A, Herrin J, Dhruva SS, Desai NR, Davis BR, Krumholz HM, Spatz ES (2019). Disparities in socioeconomic context and association with blood pressure control and cardiovascular outcomes in ALLHAT. JAHA.

[4] Carlsson AC, Li X, Holzmann MJ, Ärnlöv J, Wändell P, Gasevic D, Sundquist J, Sundquist K (2017). Neighborhood socioeconomic status at the age of 40 years and ischemic stroke before the age of 50 years: a nationwide cohort study from Sweden. Int J Stroke.

[5] Shin J, Jung M, Kwon CH (2021). Disparities in mortality and cardiovascular events by income and blood pressure levels among patients with hypertension in South Korea. JAHA.

[6] Muntner P, Hardy ST, Fine LJ, Jaeger BC, Wozniak G, Levitan EB, Colantonio LD (2020). Trends in blood pressure control among US adults with hypertension, 1999–2000 to 2017–2018. JAMA.

[7] Aggarwal R, Chiu N, Wadhera RK, Moran AE, Raber I, Shen C, Yeh RW, Kazi DS (2021). Racial/ethnic disparities in hypertension prevalence, awareness, treatment, and control in the United States, 2013 to 2018. Hypertension.

[8] Musemwa N, Gadegbeku CA (2017). Hypertension in African Americans. Curr Cardiol Rep.

[9] Samanic CM, Barbour KE, Liu Y, Wang Y, Fang J, Lu H, Schieb L, Greenlund KJ (2020). Prevalence of self-reported hypertension and antihypertensive medication use by county and rural-urban classification — United States, 2017. MMWR Morb Mortal Wkly Rep.

[10] Schutte AE, Srinivasapura Venkateshmurthy N, Mohan S, Prabhakaran D (2021). Hypertension in low- and middle-income countries. Circ Res.

[11] Singh GK, Siahpush M, Liu L, Allender M (2018). Racial/ethnic, nativity, and sociodemographic disparities in maternal hypertension in the United States, 2014–2015. Int J Hypertens.

[12] Ford ND, Cox S, Ko JY, Ouyang L, Romero L, Colarusso T, Ferre CD, Kroelinger CD, Hayes DK, Barfield WD (2022). Hypertensive disorders in pregnancy and mortality at delivery hospitalization — United States, 2017–2019. MMWR Morb Mortal Wkly Rep.

[13] Harris M, Henke C, Hearst M, Campbell K (2020). Future directions: analyzing health disparities related to maternal hypertensive disorders. J Pregnancy.

[14] Miguel E, Da S, Lopes SO, Araújo SP, Priore SE, Alfenas R de CG, Hermsdorff HHM. Association between food insecurity and cardiometabolic risk in adults and the elderly: a systematic review. J Glob Health. 2020;10:020402. 10.7189/jogh.10.020402 PMC7568919 33110569

[15] Seligman HK, Laraia BA, Kushel MB (2010). Food insecurity is associated with chronic disease among low-income NHANES participants. J Nutr.

[16] Venci BJ, Lee S-Y (2018). Functional limitation and chronic diseases are associated with food insecurity among U.S. adults. Ann Epidemiol.

[17] Chang TE, Ritchey MD, Park S, Chang A, Odom EC, Durthaler J, Jackson SL, Loustalot F (2019). National rates of nonadherence to antihypertensive medications among insured adults with hypertension, 2015. Hypertension.

[18] Elliott WJ (2009). Improving outcomes in hypertensive patients: focus on adherence and persistence with antihypertensive therapy. J Clin Hypertens (Greenwich).

[19] Lin M-Y, Weng W-S, Apriliyasari RW, VAN Truong P, Tsai P-S. Effects of patient activation intervention on chronic diseases: a meta-analysis. J Nurs Res. 2020;28:e116. 10.1097/jnr.0000000000000387 32649394

[20] Fontil V, Pacca L, Bellows BK, Khoong E, McCulloch CE, Pletcher M, Bibbins-Domingo K (2022). Association of differences in treatment intensification, missed visits, and scheduled follow-up interval with racial or ethnic disparities in blood pressure control. JAMA Cardiol.

[21] Holt HK, Gildengorin G, Karliner L, Fontil V, Pramanik R, Potter MB (2022). Differences in hypertension medication prescribing for Black Americans and their association with hypertension outcomes. J Am Board Fam Med.

[22] Cooper-DeHoff RM, Fontil V, Carton T (2021). Tracking blood pressure control performance and process metrics in 25 US health systems: the PCORnet blood pressure control laboratory. J Am Heart Assoc.

[23] Fontil V, Gupta R, Moise N, Chen E, Guzman D, McCulloch CE, Bibbins-Domingo K (2018). Adapting and evaluating a health system intervention from Kaiser Permanente to improve hypertension management and control in a large network of safety-net clinics. Circ Cardiovasc Qual Outcomes.

[24] Carter BL, Rogers M, Daly J, Zheng S, James PA (2009). The potency of team-based care interventions for hypertension: a meta-analysis. Arch Intern Med.

[25] Bui-Duy M-K, Wong S, Lam R, Karliner LS (2019). Development of a multistep hypertension quality improvement program in an academic general medicine practice. Journal for healthcare quality : official publication of the National Association for Healthcare Quality.

[26] Hebert PL, Sisk JE, Tuzzio L, Casabianca JM, Pogue VA, Wang JJ, Chen Y, Cowles C, McLaughlin MA (2012). Nurse-led disease management for hypertension control in a diverse urban community: a randomized trial. J Gen Intern Med.

[27] Clark CE, Smith LFP, Taylor RS, Campbell JL (2010). Nurse led interventions to improve control of blood pressure in people with hypertension: systematic review and meta-analysis. BMJ.

[28] Reid F, Murray P, Storrie M (2005). Implementation of a pharmacist-led clinic for hypertensive patients in primary care–a pilot study. Pharm World Sci.

[29] Morgado MP, Morgado SR, Mendes LC, Pereira LJ, Castelo-Branco M (2011). Pharmacist interventions to enhance blood pressure control and adherence to antihypertensive therapy: review and meta-analysis. Am J Health Syst Pharm.

[30] Khoong EC, Butler BA, Mesina O, Su G, DeFries TB, Nijagal M, Lyles CR (2021). Patient interest in and barriers to telemedicine video visits in a multilingual urban safety-net system. J Am Med Inform Assoc.

[31] Nouri S, Khoong EC, Lyles C, Karliner L (2020). Addressing equity in telemedicine for chronic disease management during the Covid-19 pandemic. NEJM Catalyst Innovations in Care Delivery.

[32] Uscher-Pines L, Sousa J, Jones M, Whaley C, Perrone C, McCullough C, Ober AJ (2021). Telehealth use among safety-net organizations in California during the COVID-19 pandemic. JAMA.

[33] Williams B, Mancia G, Spiering W (2018). 2018 ESC/ESH Guidelines for the management of arterial hypertension. Eur Heart J.

[34] Whelton PK, Carey RM, Aronow WS (2018). 2017 ACC/AHA/AAPA/ABC/ACPM/AGS/APhA/ASH/ASPC/NMA/PCNA guideline for the prevention, detection, evaluation, and management of high blood pressure in adults: a report of the American College of Cardiology/American Heart Association Task Force on Clinical Pr. Hypertension.

[35] Shimbo D, Artinian NT, Basile JN, Krakoff LR, Margolis KL, Rakotz MK, Wozniak G, On behalf of the American Heart Association and the American Medical Association (2020). Self-Measured Blood Pressure Monitoring at Home: A Joint Policy Statement From the American Heart Association and American Medical Association. Circulation.

[36] Peacock J, Diaz KM, Viera AJ, Schwartz JE, Shimbo D (2014). Unmasking masked hypertension: prevalence, clinical implications, diagnosis, correlates and future directions. J Hum Hypertens.

[37] Franklin SS, Thijs L, Hansen TW, O’Brien E, Staessen JA (2013). White-coat hypertension: new insights from recent studies. Hypertension.

[38] Shimbo D, Abdalla M, Falzon L, Townsend RR, Muntner P (2015). Role of ambulatory and home blood pressure monitoring in clinical practice: a narrative review. Ann Intern Med.

[39] Victor RG, Ravenell JE, Freeman A (2011). Effectiveness of a barber-based intervention for improving hypertension control in black men: the BARBER-1 study: a cluster randomized trial. Arch Intern Med.

[40] Yi SS, Wyatt LC, Patel S (2019). A faith-based intervention to reduce blood pressure in underserved Metropolitan New York immigrant communities. Prev Chronic Dis.

[41] Fuchs SC, de Mello RGB, Fuchs FC (2013). Home blood pressure monitoring is better predictor of cardiovascular disease and target organ damage than office blood pressure: a systematic review and meta-analysis. Curr Cardiol Rep.

[42] Uhlig K, Patel K, Ip S, Kitsios GD, Balk EM (2013). Self-measured blood pressure monitoring in the management of hypertension: a systematic review and meta-analysis. Ann Intern Med.

[43] Reboussin DM, Allen NB, Griswold ME, Guallar E, Hong Y, Lackland DT, Miller EPR, Polonsky T, Thompson-Paul AM, Vupputuri S (2018). Systematic review for the 2017 ACC/AHA/AAPA/ABC/ACPM/AGS/APhA/ASH/ASPC/NMA/PCNA guideline for the prevention, detection, evaluation, and management of high blood pressure in adults: a report of the American College of Cardiology/American Heart Association Task Force on clinical practice guidelines. Circulation.

[44] McManus RJ, Mant J, Franssen M (2018). Efficacy of self-monitored blood pressure, with or without telemonitoring, for titration of antihypertensive medication (TASMINH4): an unmasked randomised controlled trial. The Lancet.

[45] Bray EP, Holder R, Mant J, McManus RJ (2010). Does self-monitoring reduce blood pressure? Meta-analysis with meta-regression of randomized controlled trials. Ann Med.

[46] Glynn LG, Murphy AW, Smith SM, Schroeder K, Fahey T (2010). Self-monitoring and other non-pharmacological interventions to improve the management of hypertension in primary care: a systematic review. Br J Gen Pract.

[47] Agarwal R, Bills JE, Hecht TJW, Light RP (2011). Role of home blood pressure monitoring in overcoming therapeutic inertia and improving hypertension control: a systematic review and meta-analysis. Hypertension.

[48] Duan Y, Xie Z, Dong F, Wu Z, Lin Z, Sun N, Xu J (2017). Effectiveness of home blood pressure telemonitoring: a systematic review and meta-analysis of randomised controlled studies. J Hum Hypertens.

[49] Tucker KL, Sheppard JP, Stevens R (2017). Self-monitoring of blood pressure in hypertension: a systematic review and individual patient data meta-analysis. PLoS Med.

[50] •• Wall HK, Wright JS, Jackson SL, Daussat L, Ramkissoon N, Schieb LJ, Stolp H, Tong X, Loustalot F. How do we jump-start self-measured blood pressure monitoring in the United States?. Addressing barriers beyond the published literature. Am J Hypertens. 2022;35:244-255. **This review focuses on multi-level barriers to implementing SMBP monitoring in all clinical settings. While not focused on equity, many of the challenges discussed in this review are applicable to equity considerations.** 10.1093/ajh/hpab170 PMC10061272 35259238

[51] Centers for Disease Control and Prevention. Self-measured blood pressure monitoring with clinical support. In: Self-measured blood pressure monitoring with clinical support. 2021. https://www.cdc.gov/dhdsp/pubs/guides/best-practices/smbp.htm. Accessed 16 May 2022.

[52] Omboni S, McManus RJ, Bosworth HB (2020). Evidence and recommendations on the use of telemedicine for the management of arterial hypertension: an international expert position paper. Hypertension.

[53] Khoong EC, Sharma AE, Gupta K, Adler-Milstein J, Sarkar U. The abrupt expansion of ambulatory telemedicine: implications for patient safety. J Gen Intern Med. 2022;s11606–021–07329–9. 10.1007/s11606-021-07329-9 PMC8768444 35048294

[54] Omboni S (2019). Connected health in hypertension management. Front Cardiovasc Med.

[55] Syed ST, Gerber BS, Sharp LK (2013). Traveling towards disease: transportation barriers to health care access. J Community Health.

[56] Khoong EC, Olazo K, Rivadeneira NA, Thatipelli S, Barr-Walker J, Fontil V, Lyles CR, Sarkar U. Mobile health strategies for blood pressure self-management in urban populations with digital barriers: systematic review and meta-analyses. npj Digit Med. 4:114. 10.1038/s41746-021-00486-5 PMC8298448 34294852

[57] Turchin A, Goldberg SI, Shubina M, Einbinder JS, Conlin PR (2010). Encounter frequency and blood pressure in hypertensive patients with diabetes mellitus. Hypertension.

[58] King CC, Bartels CM, Magnan EM, Fink JT, Smith MA, Johnson HM (2017). The importance of frequent return visits and hypertension control among US young adults: a multidisciplinary group practice observational study. J Clin Hypertens.

[59] Khanna RR, Victor RG, Bibbins-Domingo K, Shapiro MF, Pletcher MJ (2012). Missed opportunities for treatment of uncontrolled hypertension at physician office visits in the United States, 2005 through 2009. Arch Intern Med.

[60] Uhlig K, Patel K, Concannon T, Balk E, Ratichek S, Kong Win Chang L, Iovin R. Self-measured blood pressure: future research needs. Age Healthcar Res Qual. 2012. 23617015

[61] • Hirshberg A, Sammel MD, Srinivas SK. Text message remote monitoring reduced racial disparities in postpartum blood pressure ascertainment. Am J Obst Gynecol. 2019;221:283–285. **This study found that using text messages to collect home blood pressure values reduced disparities in collection of postpartum blood pressure values between Black and non-Black patients when compared to collection of only in-clinic blood pressure values.** 10.1016/j.ajog.2019.05.011 31121137

[62] Triebwasser JE, Janssen MK, Hirshberg A, Srinivas SK (2020). Successful implementation of text-based blood pressure monitoring for postpartum hypertension. Pregnancy Hypertension.

[63] Eberly LA, Sanghavi M, Julien HM, Burger L, Chokshi N, Lewey J (2022). Evaluation of online patient portal vs text-based blood pressure monitoring among Black patients with Medicaid and Medicare insurance who have hypertension and cardiovascular disease. JAMA Netw Open.

[64] Boulware LE, Ephraim PL, Hill-Briggs F (2020). Hypertension self-management in socially disadvantaged African Americans: the achieving blood pressure control together (ACT) randomized comparative effectiveness trial. J GEN INTERN MED.

[65] • Cummings DM, Adams A, Halladay J, et al. Race-specific patterns of treatment intensification among hypertensive patients using home blood pressure monitoring: analysis using defined daily doses in the heart healthy lenoir study. Ann Pharmacoth. 2019;53:333–340. **This study describes the differential impact of home blood pressure monitoring on medication intensification with an explicit evaluation of differences between Black and white participants. It found no differences in medication intensification between Black and white participants.** 10.1177/1060028018806001 PMC7433691 30282468

[66] Pekmezaris R, Schwartz RM, Taylor TN (2016). A qualitative analysis to optimize a telemonitoring intervention for heart failure patients from disparity communities. BMC Med Inform Decis Mak.

[67] Skolarus LE, Cowdery J, Dome M (2018). Reach out churches: a community-based participatory research pilot trial to assess the feasibility of a mobile health technology intervention to reduce blood pressure among African Americans. Health Promot Pract.

[68] Still CH, Margevicius SP, Wright JT, Ruksakulpiwat S, Moore SM (2021). A pilot study evaluating the effects of a technology-based and positive psychological training intervention on blood pressure in African Americans with hypertension. J Prim Care Community Health.

[69] Wenger NK, Williams OO, Parashar S (2019). SMARTWOMAN™: Feasibility assessment of a smartphone app to control cardiovascular risk factors in vulnerable diabetic women. Clin Cardiol.

[70] • Yi SS, Tabaei BP, Angell SY, Rapin A, Buck MD, Pagano WG, Maselli FJ, Simmons A, Chamany S. Self-blood pressure monitoring in an urban, ethnically diverse population: a randomized clinical trial utilizing the electronic health record. Circ Cardiovasc Qual Out. 2015;8:138–45. **This is one of the earliest studies focused on implementation of SMBP monitoring in community health centers. It found that SMBP monitoring did not improve blood pressure control over usual care in a primarily Hispanic and uninsured population.** 10.1161/CIRCOUTCOMES.114.000950 PMC4366280 25737487

[71] Zha P, Qureshi R, Porter S, Chao YY, Pacquiao D, Chase S, O’Brien-Richardson P. Utilizing a mobile health intervention to manage hypertension in an underserved community. West J Nurs Res. 2019;193945919847937. 10.1177/0193945919847937 31057081

[72] Chandler J, Sox L, Kellam K, Feder L, Nemeth L, Treiber F (2019). Impact of a culturally tailored mHealth medication regimen self-management program upon blood pressure among hypertensive Hispanic adults. Int J Environ Res Public Health.

[73] Kim MT, Han H-R, Hedlin H, Kim J, Song HJ, Kim KB, Hill MN (2011). Teletransmitted monitoring of blood pressure and bilingual nurse counseling-sustained improvements in blood pressure control during 12 months in hypertensive Korean Americans: teletransmitted monitoring. The Journal of Clinical Hypertension.

[74] • Park S, Kum H-C, Morrisey MA, Zheng Q, Lawley MA. Adherence to telemonitoring therapy for medicaid patients with hypertension: case study. J Med Internet Res. 2021;23:e29018. **This study with 800+ Medicaid patients focused on adherence to a blood pressure telemonitoring interventions and found high rates of nonadherence even with reminder phone calls underscoring challenges of implementation this patient population.** 10.2196/29018 PMC8453343 34486977

[75] Clark D, Woods J, Zhang Y, Chandra S, Summers RL, Jones DW (2021). Home blood pressure telemonitoring with remote hypertension management in a rural and low-income population. Hypertension.

[76] • Cené CW, Halladay JR, Gizlice Z et al. A multicomponent quality improvement intervention to improve blood pressure and reduce racial disparities in rural primary care practices. J Clin Hypertens. 2017;19:351–360. **This study describes a multicomponent practice-based real-world effort to improve blood pressure control among six primary care practices in rural North Carolina with an explicit evaluation of the impact on disparities in blood pressure outcome between Black and white participants. They found no differences in impact between Black and white participants.** 10.1111/jch.12944 PMC8031107 27886435

[77] • Roy D, Meador M, Sasu N, Whelihan K, Lewis JH. Are community health center patients interested in self-measured blood pressure monitoring (SMBP) – and can they do it?. IBPC Volume. 2021;14:19–29. **This study surveyed patients and clinicians in nine community health centers and three states about a protocol for SMBP monitoring to assess knowledge, engagement, and experience with SMBP monitoring. It provides both quantitative and qualitative data that identifies barriers, facilitators, challenges, and success in real-world implementation of SMBP monitoring in community health centers.** 10.2147/IBPC.S285007 PMC7886240 33603456

[78] Sterling MR, Echeverría SE, Commodore-Mensah Y, Breland JY, Nunez-Smith M. Health equity and implementation science in heart, lung, blood, and sleep-related research: emerging themes from the 2018 Saunders-Watkins leadership workshop. Circ Cardiovas Qual Out. 2019;12:e005586. 10.1161/CIRCOUTCOMES.119.005586 PMC6812546 31610713

[79] • Ostchega Y, Zhang G, Kit BK, Nwankwo T. Factors associated with home blood pressure monitoring among US adults: National Health and Nutrition Examination Survey, 2011–2014. Am J Hypertens. 2000;30:1126–1132. **This study used national survey data to identify factors associated with home blood pressure monitoring and found that clinician recommendation to engage in SMBP monitoring significantly improved frequency of SMBP monitoring.** 10.1093/ajh/hpx101 PMC9880871 28633432

[80] National Institute on Minority Health and Health Disparities. National Institute on Minority Health and health disparities research framework. In: NIMHD Research Framework. 2017. https://nimhd.nih.gov/researchFramework. Accessed 13 Jun 2022.

[81] Alvidrez J, Castille D, Laude-Sharp M, Rosario A, Tabor D (2019). The National Institute on Minority Health and Health Disparities Research Framework. Am J Public Health.

[82] Lyles CR, Aulakh V, Jameson W, Schillinger D, Yee H, Sarkar U (2014). Innovation and transformation in California’s safety net health care settings: an inside perspective. American journal of medical quality : the official journal of the American College of Medical Quality.

[83] Veinot TC, Mitchell H, Ancker JS (2018). Good intentions are not enough: how informatics interventions can worsen inequality. Journal of the American Medical Informatics Association : JAMIA.

[84] Committee on Improving the Representation of Women and Underrepresented Minorities in Clinical Trials and Research, Committee on Women in Science, Engineering, and Medicine, Policy and Global Affairs, National Academies of Sciences, Engineering, and Medicine. Improving representation in clinical trials and research: building research equity for women and underrepresented groups. 2000;26479.

[85] Jackson SL, Ayala C, Tong X, Wall HK (2019). Clinical implementation of self-measured blood pressure monitoring, 2015–2016. Am J Prev Med.

[86] Broderick A, Steinmetz V, Benzinou M, Carroll S, Helms. Remote patient monitoring in the safety net: what payers and providers need to know. 2021. https://www.chcf.org/wp-content/uploads/2021/07/RemotePatientMonitoringSafetyNetNeedKnow.pdf.

[87] NYU Langone Health. Actions to decrease disparities in risk and engage in shared support for blood pressure control (ADDRESS-BP) in Blacks. 2022. https://clinicaltrials.gov/ct2/show/NCT05208450. Accessed 22 Jun 2022.

[88] •• Meador M, Hannan J, Roy D, Whelihan K, Sasu N, Hodge H, Lewis JH. Accelerating use of self-measured blood pressure monitoring (SMBP) through clinical-community care models. J Community Health. 2021;46:127–138. **This paper describes a model to accelerate adoption of SMBP by encouraging collaborations between health centers, local health departments, and local YMCAs in three states. Collaborations with community-based organizations have been found to be effective at improving chronic disease management in historically excluded populations.** 10.1007/s10900-020-00858-0 PMC7755231 32564288

[89] Lyles CR, Wachter RM, Sarkar U (2021). Focusing on digital health equity. JAMA.

[90] Lyles C, Nguyen OK, Khoong E, Aguilera A, Sarkar U. Multi-level determinants of digital health equity: a literature synthesis to advance the field. Ann Rev Publ Health. 2022. 10.1146/annurev-publhealth-071521-023913 PMC10329412 36525960

[91] McLeroy KR, Bibeau D, Steckler A, Glanz K (1988). An ecological perspective on health promotion programs. Health Educ Q.

[92] Fu SN, Dao MC, Wong CKH, Cheung BMY (2020). The association of health literacy with high-quality home blood pressure monitoring for hypertensive patients in outpatient settings. Int J Hypertens.

[93] America’s health care safety net: intact but endangered. 2000. 10.17226/9612 25077222

[94] Adler-Milstein J, Nong P (2019). Early experiences with patient generated health data: health system and patient perspectives. J Am Med Inform Assoc.

[95] Adler-Milstein J, Holmgren AJ, Kralovec P, Worzala C, Searcy T, Patel V (2017). Electronic health record adoption in US hospitals: the emergence of a digital “advanced use” divide. Journal of the American Medical Informatics Association : JAMIA.

[96] Khoong EC, Cherian R, Rivadeneira NA, Gourley G, Yazdany J, Amarnath A, Schillinger D, Sarkar U (2018). Accurate measurement in California’s safety-net health systems has gaps and barriers. Health Aff.

[97] Friedberg MW, Reid RO, Timbie JW, Setodji C, Kofner A, Weidmer B, Kahn K (2017). Federally qualified health center clinicians and staff increasingly dissatisfied with workplace conditions. Health Aff.

[98] Lyles C, Aguilera A, Nguyen O, Sarkar U. Bridging the digital health divide: how designers can create more inclusive digital health tools. California Health Care Foundation, Oakland, CA. 2022.

[99] Sarkar U, Lisker S, Lyles CR. How to narrow the digital divide in U.S. health care. Harv Bus Rev. 2021.

[100] Schaefer KR, Fyfe-Johnson AL, Noonan CJ (2021). Home blood pressure monitoring devices: device performance in an Alaska native and American Indian population. J Aging Health.

[101] Ganti V, Carek AM, Jung H, Srivatsa AV, Cherry D, Johnson LN, Inan OT (2021). Enabling wearable pulse transit time-based blood pressure estimation for medically underserved areas and health equity: comprehensive evaluation study. JMIR Mhealth Uhealth.

[102] Nouri SS, Adler-Milstein J, Thao C, Acharya P, Barr-Walker J, Sarkar U, Lyles C (2020). Patient characteristics associated with objective measures of digital health tool use in the United States: a literature review. Journal of the American Medical Informatics Association : JAMIA.

[103] Curran GM, Bauer M, Mittman B, Pyne JM, Stetler C (2012). Effectiveness-implementation hybrid designs: combining elements of clinical effectiveness and implementation research to enhance public health impact. Med Care.

[104] Fawzy A, Wu TD, Wang K, Robinson ML, Farha J, Bradke A, Golden SH, Xu Y, Garibaldi BT (2022). Racial and ethnic discrepancy in pulse oximetry and delayed identification of treatment eligibility among patients with COVID-19. JAMA Intern Med.

[105] Feathers T. Google’s new dermatology app wasn’t designed for people with darker skin. Vice. 2021.

[106] Hirshberg A, Downes K, Srinivas S (2018). Comparing standard office-based follow-up with text-based remote monitoring in the management of postpartum hypertension: a randomised clinical trial. BMJ Qual Saf.

[107] Johns Hopkins University. A cardiometabolic health program linked with clinical-community support and mobile health telemonitoring to reduce health disparities. 2022a. https://clinicaltrials.gov/ct2/show/NCT05321368. Accessed 22 Jun 2022.

[108] Johns Hopkins University. Home blood pressure telemonitoring LINKED with community health workers to improve blood pressure. 2022b. https://clinicaltrials.gov/ct2/show/NCT05180045. Accessed 22 Jun 2022.

[109] Dillard D. Home blood pressure monitoring intervention for self-management of high blood pressure among Alaska native people. 2022. https://clinicaltrials.gov/ct2/show/NCT03872856. Accessed 22 Jun 2022.

[110] Still C. Optimizing technology to improve medication adherence and blood pressure control. 2022. https://clinicaltrials.gov/ct2/show/NCT05293756. Accessed 22 Jun 2022

[111] Brewer LC. Patient-provider-community health worker integrated care model: use of an innovative mobile health intervention to improve hypertension among African-Americans. 2022. https://clinicaltrials.gov/ct2/show/NCT04554147. Accessed 22 Jun 2022.

[112] Sharrief AZ. Video-based intervention to reduce treatment and outcome disparities in adults living with stroke or transient ischemic attack (VIRTUAL). 2022. https://clinicaltrials.gov/ct2/show/NCT05264298. Accessed 22 Jun 2022. 10.1186/s13063-025-09003-5 PMC12344908 40796883

